# Phase I study of high-dose ascorbic acid with mFOLFOX6 or FOLFIRI in patients with metastatic colorectal cancer or gastric cancer

**DOI:** 10.1186/s12885-019-5696-z

**Published:** 2019-05-16

**Authors:** Feng Wang, Ming-Ming He, Zi-Xian Wang, Su Li, Ying Jin, Chao Ren, Si-Mei Shi, Bing-Tian Bi, Shuang-Zhen Chen, Zhi-Da Lv, Jia-Jia Hu, Zhi-Qiang Wang, Feng-Hua Wang, De-Shen Wang, Yu-Hong Li, Rui-Hua Xu

**Affiliations:** 1 State Key Laboratory of Oncology in South China, Collaborative Innovation Center for Cancer Medicine, Sun Yat-sen University Cancer Center, Guangzhou, 510060 People’s Republic of China; 2 Department of Medical Oncology, Sun Yat-sen University Cancer Center, Guangzhou, 510060 China; 3 Department of Clinical Trial Center, Sun Yat-sen University Cancer Center, Guangzhou, 510060 China

**Keywords:** Ascorbic acid, Metastatic colorectal cancer, Metastatic gastric cancer, Recommended phase 2 dose; chemotherapy

## Abstract

**Background:**

Preclinical studies suggest synergistic effectiveness of ascorbic acid (AA, vitamin C) and cytotoxic agents in gastrointestinal malignancies. This phase 1 study aimed to establish the maximum tolerated dose (MTD) and recommended phase 2 dose (RP2D) of AA combined with mFOLFOX6 or FOLFIRI regimens in patients with metastatic colorectal cancer (mCRC) or gastric cancer (mGC).

**Methods:**

In the dose-escalation phase, patients received AA (0.2–1.5 g/kg, 3-h infusion, once daily, days 1–3) with mFOLFOX6 or FOLFIRI in a 14-day cycle until the MTD was reached. In the speed-expansion phase, AA was administered at the MTD or at 1.5 g/kg if the MTD was not reached at a fixed rate of 0.6, 0.8 or 1 g/min. Pharmacokinetics and preliminary efficacy were also assessed.

**Results:**

Thirty-six patients were enrolled. The MTD was not reached. The RP2D was established as AA at 1.5 g/kg/day, days 1–3, with mFOLFOX6 or FOLFIRI. No dose-limiting toxicity (DLT) was detected during dose escalation. The most common treatment-emergent adverse events (TRAEs) were sensory neuropathy (50%), nausea (38.9%), vomiting (36.1%) and neutropenia (27.8%). Grade 3–4 TRAEs were neutropenia (13.9%), sensory neuropathy (2.8%), vomiting (2.8%), diarrhea (2.8%) and leukopenia (2.8%). AA exposure was dose-proportional. The objective response rate was 58.3%, and the disease control rate was 95.8%. No difference in efficacy was found between mCRC patients with wild-type RAS/BRAF and mutant RAS or BRAF.

**Conclusions:**

The favorable safety profile and preliminary efficacy of AA plus mFOLFOX6/FOLFIRI support further evaluation of this combination in mCRC or mGC.

**Trial registration:**

ClinicalTrial.gov Identifier: NCT02969681.

**Electronic supplementary material:**

The online version of this article (10.1186/s12885-019-5696-z) contains supplementary material, which is available to authorized users.

## Background

The role of ascorbic acid (AA, vitamin C) in both cancer prevention and treatment has been controversial [1]. Epidemiological evidence suggests that ingestion of AA-rich foods might be associated with reduced cancer incidence [2]. However, this was not confirmed in randomized intervention trials [3, 4]. In the 1970s, a retrospective study by Ewan Cameron and Linus Pauling reported that high doses of intravenous and oral AA increased the average survival of advanced cancer patients compared with that of controls [5, 6]. However, two subsequent placebo-controlled randomized clinical trials investigating the same dose of oral AA in patients with advanced cancer were both negative, leading to decreased interest in the use of AA in cancer treatment [7, 8].

Recent preclinical and clinical studies have regenerated interest in the potential anticancer effects of AA [9–11]. Preclinical studies have reported that human colorectal cancer cells harboring KRAS or BRAF mutations are selectively killed when exposed to high levels of AA [12]. Treatment with AA also suppresses colony formation and leukemia progression in primary human leukemia patient-derived xenografts (PDXs) [13]. Clinical studies have shown that high (millimolar) plasma AA concentrations, which are selectively cytotoxic to many neoplastic cell lines, can only be achieved with intravenous infusion rather than oral administration [14–16].

Although the high AA monotherapy i.v. dose was well-tolerated, it failed to demonstrate anticancer activity in patients with previously treated advanced malignancies [17]. However, preclinical studies have suggested a synergistic effect between cytotoxic agents and AA, in which high concentrations of this redox-active compound might modify either the treatment response or toxicity [11, 17]. Moreover, existing evidence suggests that high-dose AA can safely be given alongside cytotoxic agents, such as gemcitabine, paclitaxel, and carboplatin [18, 19].

The present phase I study (ClinicalTrial.gov Identifier: NCT02969681) aimed to assess the safety, pharmacokinetic (PK) profile, and preliminary efficacy of AA in combination with mFOLFOX6 or FOLFIRI regimens in Chinese patients with metastatic colorectal cancer (mCRC) or gastric cancer (mGC).

## Methods

### Study design

**Fig. 1:**
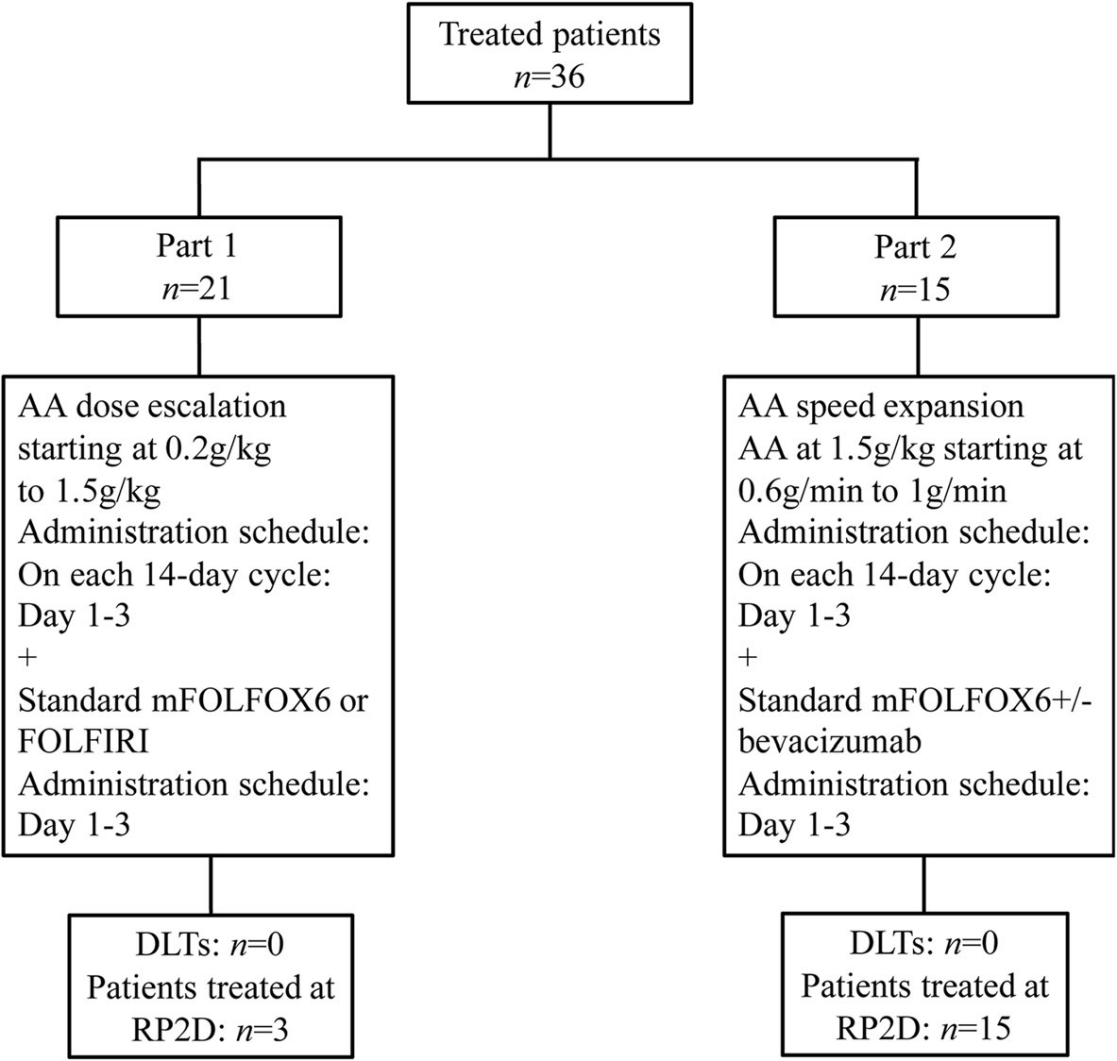
Study design. Ascorbic acid was administered in both study parts with 5-FU continuous infusion. Standard mFOLOX6 comprised a standard dose of 85 mg/m^2^ oxaliplatin on day 1 + leucovorin at 400 mg/m^2^ (2-h infusion during oxaliplatin administration) + 5FU at 400 mg/m^2^ (bolus immediately following irinotecan administration) and 2400 mg/m^2^ (46-h continuous infusion), every 2 weeks. Standard FOLFIRI consisted of a standard dose of irinotecan at 180 mg/m2 (90-min infusion) + leucovorin 400 mg/m^2^ (2-h infusion during irinotecan administration) + 5-FU at 400 mg/m2 (bolus immediately following irinotecan administration) and 2400 mg/m^2^ (46-h continuous infusion), every 2 weeks. In part 1, AA was administered once daily at 180 min (part 1) or at a fixed rate of 0.6, 0.8 and 1 g/min (part 2) for three consecutive days with continuous 5-FU infusion in a 14-day cycle

This phase 1 open-label, single-center, dose-escalation, and speed-expansion study evaluated AA in combination with mFOLFOX6 or FOLFIRI in patients with mCRC or mGC. The primary objective was to evaluate the safety profile and determine the maximum tolerated dose (MTD) and the recommended phase 2 dose (RP2D) of AA when coadministered with mFOLFOX6 or FOLFIRI. Secondary objectives were to assess the PK profile and preliminary anti-tumor activity of AA in combination with mFOLFOX6 or FOLFIRI. Patients received AA and mFOLFOX6 or FOLFIRI in 14-day cycles. AA was administered on days 1–3 with chemotherapy (Fig. 1). The trial was registered with the ClinicalTrials.gov registry on November 21, 2016 and was approved by the Independent Institute Research Ethics Committee at the Sun Yat-sen University Cancer Center prior to initiation. This trial was conducted in accordance with the Declaration of Helsinki, the guidelines for Good Clinical Practice, the European Union Clinical Trial Directive, and local regulations. All participants provided written informed consent. This report adheres to the CONSORT guidelines.

### Patients

Patients with histologically confirmed mCRC or mGC who were scheduled to receive mFOLFOX6 or FOLFIRI as first-line or second-line chemotherapy regimens without a targeted therapy agent were enrolled in part 1. Patients with histologically confirmed mCRC who were scheduled to receive mFOLFOX6 with or without bevacizumab as the first-line therapy were enrolled in part 2. In addition, patients were required to be ≥18 years and ≤ 75 years of age; have an Eastern Cooperative Oncology Group (ECOG) performance status of 0–1; a G6PD status greater than the lower limit of normal; a life expectancy of at least 12 weeks; and adequate hematologic function (ANC ≥ 1500/mm3; hemoglobin > 8 g/dL; platelet ≥100,000/mm3), renal function [creatinine ≤1.5× upper limit of normal (ULN); if the creatinine level was elevated but ≤1.5× the ULN, a 24-h creatinine clearance would be obtained, and creatinine clearance was required to be ≥50 ml/min (calculated according to Cockroft and Gault)] and hepatic function [transaminase (AST/ALT) ≤ 2.5× ULN and bilirubin levels ≤1.5× ULN without liver metastasis; transaminase (AST/ALT) ≤ 5× ULN and bilirubin levels ≤1.5× ULN with liver metastasis]. Women of childbearing potential needed to confirm a negative pregnancy test and practice effective contraception during the study. Written informed consent was required.

The exclusion criteria were failure of both oxaliplatin- and irinotecan-based regimens; surgery (excluding diagnostic biopsy) or irradiation within 3 weeks prior to study entry; administration of any investigational drug or agent/procedure, i.e., participation in another trial within 4 weeks before beginning treatment; concurrent chronic systemic immune therapy, chemotherapy, radiation therapy (palliative radiation therapy allowed) or hormone therapy not indicated in the study protocol; brain metastasis (known or suspected); pregnant or lactating women; other uncontrolled concomitant illness, including serious uncontrolled intercurrent infection; known allergy or any other adverse reaction to any of the drugs or to any related compound; previous (within 5 years) or concurrent malignancies at other sites with the exception of surgically cured or adequately treated carcinoma in situ of the cervix and basal cell carcinoma of the skin; patients who were on strong inducers of CYP3A4, including but not limited to aminoglutethimide, bexarotene, bosentan, carbamazepine, dexamethasone, efavirenz, fosphenytoin, griseofulvin, modafinil, nafcillin, nevirapine, oxcarbazepine, phenobarbital, phenytoin, primidone, rifabutin, rifampin, rifapentine, and St. John’s wort; a medical, social or psychological condition, which in the opinion of the investigator would not permit the patient to complete the study or sign a meaningful informed consent; organ allograft requiring immunosuppressive therapy; and patients with HIV infection.

### Treatment schedule

In the dose-escalation phase (part 1), the starting dose of AA (3-h infusion, once daily) was 0.2 g/kg. Dose escalation of AA was performed according to the standard 3 + 3 dose-finding scheme, in which up to three additional patients were enrolled at any dose level where a dose-limiting toxicity (DLT) was recorded. Subsequent doses were 0.4 g/kg, 0.6 g/kg, 0.8 g/kg, 1.0 g/kg, 1.2 g/kg and 1.5 g/kg until the MTD was reached. The MTD or RP2D were defined as the dose levels at which fewer than 33% of the enrolled patients experienced DLT. DLTs were defined as any grade 3 or 4 hematologic toxicity lasting 1 week or longer or as any grade 3 or 4 nonhematologic toxicity upon completion of one 2-week treatment cycle (14 days). Manageable nausea and vomiting, fatigue, anorexia, anemia, alopecia, alkaline phosphatase changes, fever without neutropenia, and local reactions were not included in the determination of DLT. Patients not completing the first cycle for reasons other than DLTs were considered not evaluable and replaced.

The chemotherapy backbone (mFOLFOX6 or FOLFIRI) was chosen at the investigators’ discretion. The chemotherapy regimens were as follows: mFOLOX6 comprised oxaliplatin 85 mg/m^2^ on day 1 concurrent with leucovorin 400 mg/m^2^, a bolus of 5FU 400 mg/m^2^, followed by infusion of 5FU at 2400 mg/m^2^ over 46 h, every 2 weeks; FOLFIRI comprised irinotecan CPT-11180 mg/m^2^ on day 1 concurrent with leucovorin 400 mg/m^2^, a bolus of 5FU 400 mg/m^2^, followed by infusion of 5FU 2400 mg/m^2^ over 46 h, every 2 weeks. For patients with mCRC who chose to receive bevacizumab, the dose was 5 mg/kg every 2 weeks. In part 1, AA was administered once daily for 180 min for three consecutive days with mFOLFOX6 or FOLFIRI in a 14-day cycle.

In the speed-expansion phase (part 2), AA was administered at the MTD or 1.5 g/kg, if the MTD was not reached, once daily at a fixed rate of 0.6, 0.8 and 1 g/min (five patients each) for three consecutive days with mFOLFOX6 in a 14-day cycle.

The treatment continued until 12 cycles, disease progression, unmanageable toxic effects, or withdrawal of consent. For patients who suffered from greater than grade 2 peripheral neurotoxicity, oxaliplatin treatment was stopped.

### Safety, pharmacokinetic, and efficacy assessments

All treated patients were assessed for safety, including adverse events, complete blood count, laboratory assessments, vital signs, electrocardiogram, and physical examination. Adverse events were defined and graded according to the NCI Common Terminology Criteria for Adverse Events (CTCAE, v4.03) and were recorded from enrollment until 3 weeks after the last dose. Pre- and postdose 5-ml blood samples were collected at prespecified time points in cycle 1 during and after infusions. Symptoms or side-effects that were associated with rapid infusion of high-osmolarity solution or treatment-related adverse events that were possibly attributed to high-dose AA were defined as DLT. [17]

Blood samples were taken immediately before infusion, mid-infusion, at the end-point, and at 1, 3, 6, and 12 h after the end-point. Patient plasma samples were placed in individual tubes, protected from light, and then frozen at − 80 °C until the assays were carried out. Plasma samples for PK analyses were obtained on the first and third day of the first week of dosing. AA was measured in 3-ml blood samples at the indicated times by LC-MS/MS. Briefly, plasma samples were diluted 100-times with normal saline containing 0.05% EDTA. An aliquot (100 μl) of the diluted plasma was extracted by adding 400 μl of acetonitrile with an internal standard. Samples were then vortexed for 1 min and centrifuged at 12000 rpm for 10 min at 4 °C. A 2-μl sample was injected into the high-performance liquid chromatography system using a temperature-controlled autosampling device maintained at 4 °C. Separation of analytes was achieved using a PLRP-S column (Agilent, 150 × 2.1 mm, 8 μm, 1000 Å). The mobile phase used for chromatographic separation consisted of water containing 0.1% formic acid as eluent A and acetonitrile as eluent B. The analytes were separated in a gradient at a flow rate of 0.4 ml/min. The column effluent was monitored using an AB Sciex 5500 system. The samples were analyzed using an electrospray probe in the negative ionization mode operating at the m/z for AA and that of the internal standard m/z with a spray voltage of − 4500 V. A good linearity was demonstrated within the range of 5–50 μg/mL. The PK parameters were determined by WinNonlin software, including the maximal mM drug-concentration in plasma (Cmax), the area under the drug-concentration curve (AUC) from infusion start to extrapolated infinite time, clearance, and noncompartmental terminal elimination rate.

Computed tomography or MRI were performed within 4 weeks before treatment initiation, after every 3 cycles, and 3 weeks after the final dose. Tumor response was assessed using Response Evaluation Criteria in Solid Tumors (RECIST) v. 1.1.

### Statistical analyses

Descriptive statistics were used for analysis of the safety, pharmacokinetics, and tumor response data. The study population for safety included patients who received at least one dose of the study medication. The tumor response was assessed in all patients who received at least one dose of the study medication, had measurable disease at the baseline per RECIST v1.1 as assessed by central review, and had at least one post-baseline scan. Progression-free survival (PFS) was measured from the date of first exposure to the study drugs to the earliest date of disease progression or death from any cause, whichever came first.

## Results

### Patient characteristics and treatment exposure

A total of 36 patients were enrolled in this phase I trial between March 18, 2017 and June 14, 2017 (Fig. 1). The demographics and baseline characteristics of the study participants are presented in Table 1. Thirty patients with mCRC and six with mGC were recruited. Among the 30 patients with mCRC, 26 patients were assayed for KRAS, NRAS and BRAF status. Among them, 10 patients carried KRAS mutations, 2 patients carried BRAF V600E mutation and 14 had wild-type RAS and BRAF. Thirty-five patients received FOLFOX6 (first-line for all) and one received FOLFIRI (second-line) as the chemotherapy backbone. Fourteen of 30 (46.7%) patients with mCRC received bevacizumab with chemotherapy and AA.

**Table 1:** Patient characteristics (*N* = 36)

Characteristics	No. (%)
Age, years	
Median (range)	53 (27–75)
< 65	28 (77.8)
≥ 65	8 (22.2)
Sex	
Male	21 (58.3)
Female	15 (41.7)
ECOG performance status	
0	2 (5.6)
1	33 (91.7)
2	1 (2.8)
Weight, kg, median (range)	57.0 (39.0–77.0)
Cancer type	
Colorectal	30 (83.3)
Gastric	6 (16.7)
Stage at diagnosis	
Synchronous metastasis	28 (77.8)
Metachronous metastasis	8 (22.2)
Prior chemotherapy	
Yes	7 (19.4)
No	29 (80.6)
Previous surgery to the primary site	
Yes	19 (52.8)
No	17 (47.2)
Add Avastin	
Yes	14 (38.9)
No	22 (61.1)
RAS and BRAF status	
KRAS mutant	10 (27.8)
BRAF V600E mutant	2 (5.6)
RAS and BRAF wild-type	14 (38.9)
Unknown	10 (27.8)

*ECOG* Eastern Cooperative Oncology Group

Overall, patients were treated with a median of 8 cycles of chemotherapy with AA (range, 1–12). At the time of the data cutoff, all patients had discontinued AA and/or the study chemotherapeutic regimens. The reasons for discontinuation of AA and/or chemotherapy included completion of 12 cycles (5 patients, 13.9%), a switch to capecitabine-based maintenance therapy (7 patients, 19.4%), surgery (4 patients, 11.1%), disease progression (11 patients, 30.6%), a loss of follow-up (5 patients, 13.9%), and abandonment of chemotherapy (4, 11.1%).

### MTD

Twenty-one patients enrolled in the dose-escalation group were evaluable for DLT. No DLT was observed at any of the doses, and the MTD was not reached. Thus, the RP2D was established as AA at 1.5 g/kg once daily for three consecutive days in combination with standard mFOLFOX6 with or without bevacizumab every 14 days. All 15 patients enrolled into the speed-escalation group were evaluated for DLT. No DLT was observed at any of the speeds.

### Safety

All 36 patients enrolled were evaluated for safety. Treatment-related adverse events (TEAEs) of any grade were recorded in 32 patients (88.9%) (Table 2). The most common all-grade TEAEs were peripheral sensory neuropathy (50.0%), nausea (38.9%), vomiting (36.1%) and neutropenia (27.8%). Grade 3 TEAEs occurred in nine (25.0%) patients, five (13.9%) of whom experienced grade 3 neutropenia, one (2.8%) experienced vomiting, one (2.8%) grade 3 diarrhea, one (2.8%) leukopenia, and one (2.8%) grade 3 peripheral sensory neuropathy. Grade 4 TEAEs occurred in one (2.8%) patient who had neutropenia. No 5 TEAEs and no serious adverse events were detected. No patients discontinued AA and/or chemotherapy due to intolerable toxicity. Among the 26 patients treated with at least 6 cycles of oxaliplatin, grade 1 sensory neuropathy occurred in nine patients (25.0%), grade 2 in eight patients (22.2%), and grade 3 in one patient (2.8%).

**Table 2:** Treatment-emergent adverse events for the study cohort (*N* = 36)

Adverse event	No (%)				
	All grades	Grade 1	Grade 2	Grade 3	Grade4
Peripheral sensory neuropathy	18 (50.0)	9 (25.0)	8 (22.2)	1 (2.8)	0 (0)
Nausea	14 (38.9)	13 (36.1)	1 (2.8)	0 (0)	0 (0)
Vomiting	13 (36.1)	8 (22.2)	4 (11.1)	1 (2.8)	0 (0)
Neutropenia	10 (27.8)	0 (0)	4 (11.1)	5 (13.9)	1 (2.8)
Abdominal pain	9 (25.0)	9 (25.0)	0 (0)	0 (0)	0 (0)
Decreased appetite	8 (22.2)	8 (22.2)	0 (0)	0 (0)	0 (0)
leukopenia	8 (22.2)	3 (8.2)	4 (11.1)	1 (2.8)	0 (0)
Diarrhea	7 (19.4)	5 (13.9)	1 (2.8)	1 (2.8)	0 (0)
Alopecia	4 (11.1)	4 (11.1)	0 (0)	0 (0)	0 (0)
Malaise	4 (11.1)	4 (11.1)	0 (0)	0 (0)	0 (0)
Fatigue	3 (8.2)	3 (8.2)	0 (0)	0 (0)	0 (0)
Hand-foot syndrome	3 (8.2)	3 (8.2)	0 (0)	0 (0)	0 (0)
Mucositis oral	3 (8.2)	3 (8.2)	0 (0)	0 (0)	0 (0)
Hemorrhage, vagina	2 (5.6)	2 (5.6)	0 (0)	0 (0)	0 (0)
Dyspnea	2 (5.6)	2 (5.6)	0 (0)	0 (0)	0 (0)
Thrombocytopenia	2 (5.6)	1 (2.8)	1 (2.8)	0 (0)	0 (0)
Anemia	2 (5.6)	1 (2.8)	1 (2.8)	0 (0)	0 (0)
Hyperpigmentation	2 (5.6)	2 (5.6)	0 (0)	0 (0)	0 (0)
Dizziness	2 (5.6)	2 (5.6)	0 (0)	0 (0)	0 (0)
Pain	2 (5.6)	2 (5.6)	0 (0)	0 (0)	0 (0)
Gingival pain	2 (5.6)	2 (5.6)	0 (0)	0 (0)	0 (0)
Fever	2 (5.6)	2 (5.6)	0 (0)	0 (0)	0 (0)
Palpitations	2 (5.6)	2 (5.6)	0 (0)	0 (0)	0 (0)
Upper respiratory tract infection	1 (2.8)	1 (2.8)	0 (0)	0 (0)	0 (0)
Skin rash	1 (2.8)	0 (0)	1 (2.8)	0 (0)	0 (0)
Hematuresis	1 (2.8)	1 (2.8)	0 (0)	0 (0)	0 (0)
Weight loss	1 (2.8)	1 (2.8)	0 (0)	0 (0)	0 (0)
Tinnitus	1 (2.8)	1 (2.8)	0 (0)	0 (0)	0 (0)
Cough	1 (2.8)	1 (2.8)	0 (0)	0 (0)	0 (0)
Epistaxis	1 (2.8)	1 (2.8)	0 (0)	0 (0)	0 (0)
Productive cough	1 (2.8)	1 (2.8)	0 (0)	0 (0)	0 (0)
Hypertension	1 (2.8)	1 (2.8)	0 (0)	0 (0)	0 (0)

### Pharmacokinetics and pharmacodynamics

**Fig. 2:**
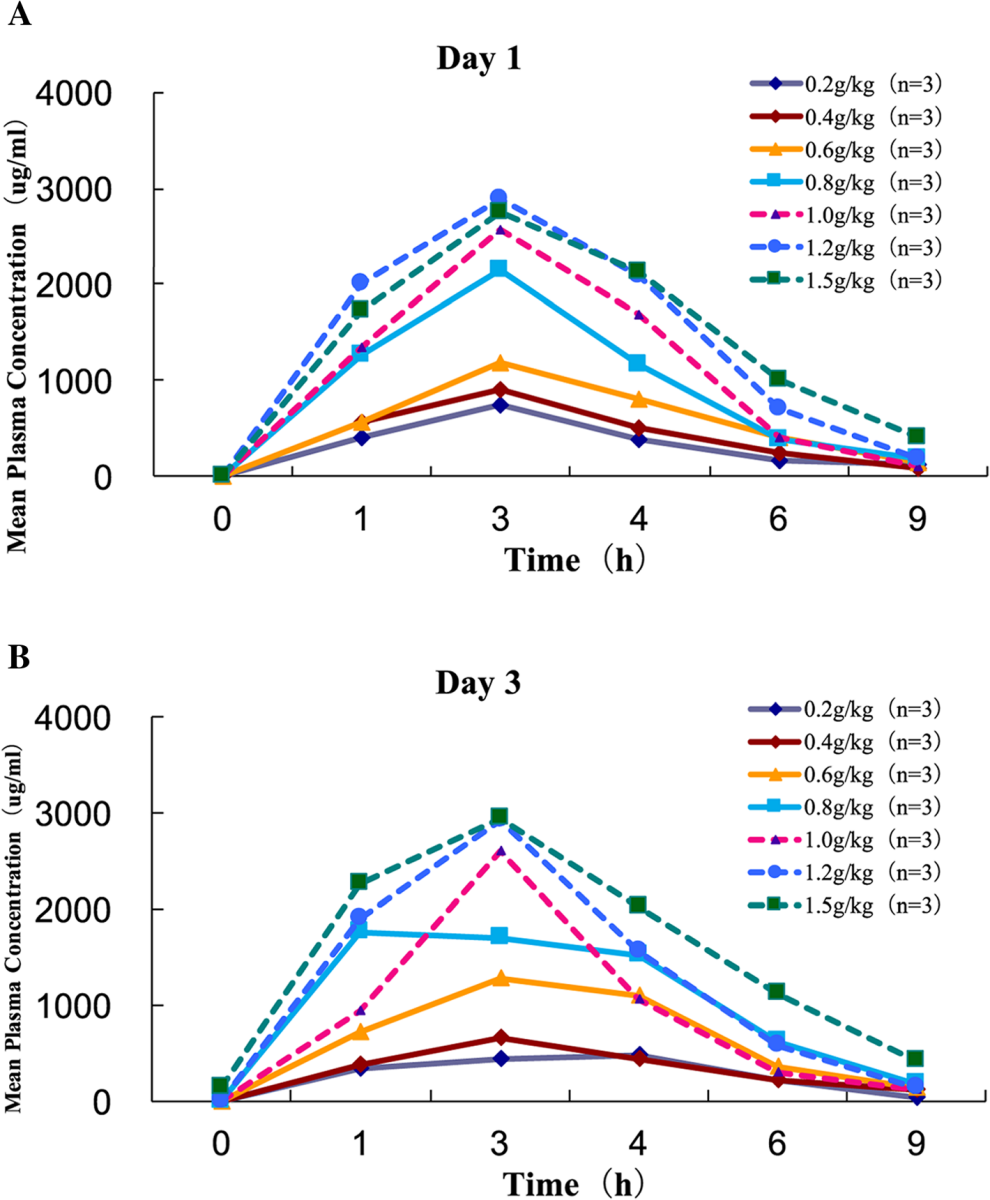
Mean plasma concentration–time curve of ascorbic acid after 0.2~1.5 g/kg administration to cancer patients (*n* = 3) on Day 1 (**a**) and Day 3 (**b**). After i.v. administration, the plasma concentrations of ascorbic acid rose gradually and peaked at 3 h. The Cmax, and AUC values of ascorbic acid displayed dose-dependent increases. Ascorbic acid concentrations in the high-dose groups remained at 10–20 mmol/L for approximately 3 h and showed no accumulation in the body during 3 daily administrations. Curves varying in linetypes and colors correspond to different administration dosages of ascorbic acid

During continuous infusion, the plasma AA concentrations rose from normal values (< 0.1 mmol/L) to concentrations that peaked at the end of the infusion (Fig. 2). The AA was eliminated by simple first-order kinetics. The AA elimination half-life (t_1/2_), clearance, C_max_, and AUC in the fixed 3-h infusion group are presented in Table 3. AA did not accumulate to any significant level during 3 consecutive daily administrations, and the t_1/2_, C_max_, and AUC values of AA for each patient did not systematically change between the first and the third treatment. The C_max_ and AUC values increased between 3.5~17.8 mmol/L and 14.9~89.5 mmol/L*h, respectively. On day 1 and day 3 of administration, the correlation coefficients (R^2^) were 0.9523 and 0.9195 for the C_max_ versus dose, and 0.9697 and 0.8657 for the AUC versus dose in the dosing range (Additional file 1: Figure S1). The t_1/2_ values (1.4~3.1 h) of AA were similar for all patients in all cohorts. However, the C_max_ and AUC appeared to reach maximum values at a dosage of 1.5 g/kg/day. Each of the three high dosages (1.0~1.5 g/kg/day) maintained AA blood levels at 10–20 mmol/L for approximately 3 h, and there were no significant differences (two-tailed Student’s t test) in the C_max_ values between day 1 and day 3 of administration for each dose. Although this effective level (10–20 mmol/L) of AA was similar between the three high dosages (1.0~1.5 g/kg/day), fixed-rate infusion trials were carried out.

**Table 3:** Pharmacokinetic values

	Unit	0.2 g/kg(*n* = 3)	0.4 g/kg(*n* = 3)	0.6 g/kg(*n* = 3)	0.8 g/kg(*n* = 3)	1 g/kg(*n* = 2)	1.2 g/kg(*n* = 3)	1.5 g/kg(*n* = 3)
D1	Cmax (mmol/l)	4.2 ± 3.9	5.1 ± 1.1	7.4 ± 1.2	12.1 ± 5.7	14.0 ± 3.9	16.4 ± 2.5	17.4 ± 7.2
	t1/2(h)	3.1 ± 1.2	1.8 ± 0.6	2.0 ± 0.2	1.9 ± 0.05	1.5 ± 0.2	1.4 ± 0.09	1.97 ± 0.8
	AUC (mmol/l *h)	21.0 ± 18.0	22.5 ± 8.8	31.5 ± 3.0	49.0 ± 15.8	55.3 ± 2.3	73.3 ± 12.3	82.3 ± 43.3
	Vz (ml)	15.3 ± 5.6	13.6 ± 1.7	14.2 ± 3.8	14.2 ± 2.4	12.7 ± 1.6	11.5 ± 1.3	14.9 ± 4.0
	Cl (ml/h)	4.1 ± 2.5	5.8 ± 2.9	4.9 ± 1.4	5.1 ± 1.0	6.0 ± 0.03	5.5 ± 0.4	5.5 ± 1.7
D3	Cmax (mmol/l)	3.5 ± 1.7	3.7 ± 0.9	7.2 ± 1.1	11.2 ± 3.3	9.5 ± 3.0	16.5 ± 1.5	17.8 ± 5.5
	t1/2(h)	1.9 ± 0.8	2.4 ± 0.9	1.7 ± 0.4	1.6 ± 0.1	1.5 ± 0.2	1.5 ± 0.06	1.98 ± 0.6
	AUC (mmol/l *h)	14.9 ± 7.4	19.0 ± 7.5	34.1 ± 3.9	54.8 ± 16.1	35.4 ± 15.8	65.5 ± 8.0	89.5 ± 52.4
	Vz (ml)	12.2 ± 7.6	21.4 ± 3.2	10.6 ± 3.6	10.9 ± 2.7	22.6 ± 7.7	13.3 ± 3.4	14.2 ± 3.6
	Cl (ml/h)	4.4 ± 1.8	6.9 ± 3.5	4.4 ± 0.6	4.6 ± 1.0	10.5 ± 5.0	6.2 ± 1.4	5.3 ± 2.1

When 1.5 g/kg of vitamin C was given during fixed-rate infusion (0.6, 0.8, 1 g/min), plasma concentrations were much higher than when the vitamin was given at a fixed infusion time (Additional file 2: Figure S2). The mean peak values from the fixed-rate administration were 25.58 mmol/L, at 10–20 mmol/L for approximately 3 h. As shown in Additional file 3: Table S1, the volumes of distribution and clearance were similar to those parameters in the fixed infusion time group. When the recommended subsequent dose was administered, the plasma concentrations of AA were maintained at 10–20 mmol/L for more than 4 h.

### Exploratory efficacy findings

**Fig. 3:**
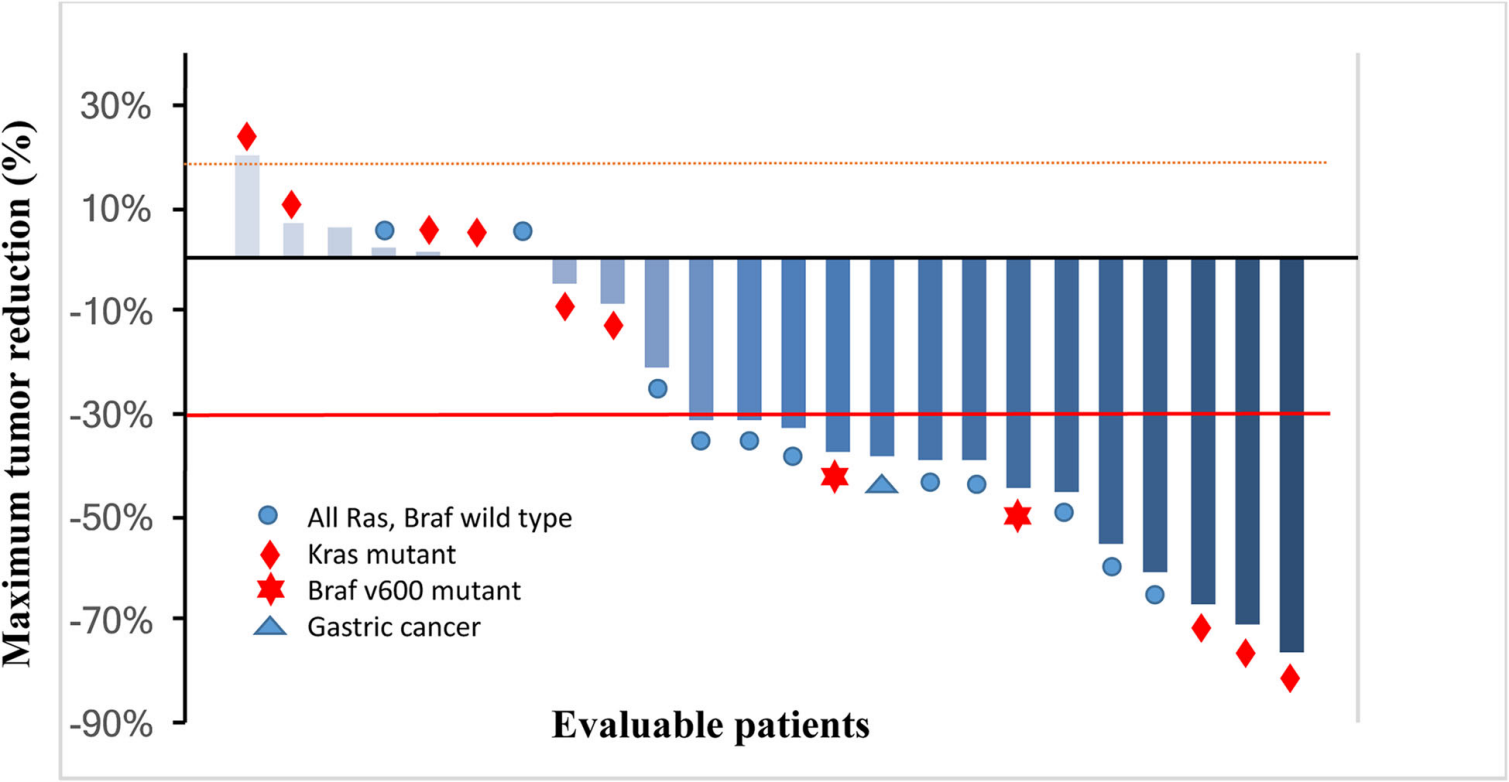
Relative change from baseline in target lesion size (at the best tumor response). Shown is the percent change in the lowest sum of the target lesions from the baseline for patients who were on treatment for 3 cycles. Twenty-three patients had documented RAS and Braf genetic alterations

Of the 36 patients enrolled, 24 (23 with mCRC and 1 with mGC) were evaluated for tumor response. The best overall responses included a partial response in fourteen patients (objective response rate, 58.3%) and stable disease in nine (37.5%), giving a disease control rate of 95.8%. Among 22 patients with mCRC receiving first-line therapy, the objective response rate was 59.1% and the disease control rate was 95.5%. Figure 3 shows the maximum percentage change from the baseline in measurable target lesions. Notably, patients with wild-type RAS/BRAF or mutant KRAS or BRAF all showed good response to the treatment. No difference in efficacy was found between mCRC patients with wild-type RAS/BRAF and mutant KRAS or BRAF according to Fisher’s test (*p* = 0.387).

With a median follow-up of 8.6 months (interquartile range, 7.4–11.4) at the cutoff date for the collection of survival data (May 15, 2018), 17 PFS events (16 disease progression and 1 death) were recorded. The median PFS for the entire cohort was 8.8 months. Of note, the PFS of the two patients with mutant BRAF was 9.6 and 5.1 months.

## Discussion

To the best of our knowledge, this is the first study investigating the safety profile and MTD for the combination of AA and chemotherapy among the East Asian population with advanced solid tumors. Our findings suggest that AA at 1.5 g/kg once daily for three consecutive days can be safely coadministered with mFOLFOX6 or FOLFIRI in a 14-day cycle, and although preliminary, this combination exhibited encouraging clinical efficacy.

The most commonly reported side effects were attributable to high-dose AA and included headache, light-headedness, dry mouth, and gastrointestinal toxicity due to rapid infusion and high osmotic load [19–21]. However, these adverse events were generally uncommon in this study. Grade 1–2 gastrointestinal toxicities with incidences of 20–40% were recorded, but their relevance to chemotherapy could not be excluded.

Previous studies have suggested that the combination of AA with chemotherapeutic agents reduced chemotherapy-associated toxicity [18, 22]. In a pilot phase 1/2a trial for stage III/IV ovarian cancer, the addition of AA to paclitaxel and carboplatin substantially decreased diverse categories of toxicity, including neurologic, dermatologic, and bone marrow toxicity, and toxicities in the renal/genitourinary and gastrointestinal systems [18]. Additionally, AA did not appear to increase the toxicity of gemcitabine, arsenic trioxide, melphalan, bortezomib, or dexamethasone [19, 23, 24]. In line with these findings, the current study showed markedly decreased all-grade and grade ≥ 3 bone marrow and gastrointestinal toxic effects compared with previous trials investigating the same chemotherapeutic regimens in mCRC or mGC. For instance, the incidence of grade ≥ 3 neutropenia was 13.9% in the current study, in contrast to approximately 30% in previous studies. [25, 26] Moreover, neurotoxicity in patients treated with at least 6 cycles of oxaliplatin was much less common than that in trials discontinuing oxaliplatin after six cycles (grade 3 neurotoxicity, 2.8% in our study versus approximately 20% in prior studies) [27, 28].

Intravenous AA administered at a dosage of 1.5 g/kg three times weekly appears to be safe and free of important toxicity in appropriately screened patients with advanced untreatable malignancies. A phase 1 trial with a fixed infusion time stopped the dose escalation at 1.5 g/kg when peak blood levels approached a plateau of 10–20 mmol/L, the level that can inhibit tumor growth in mice. Although the Cmax and AUC values for AA increased proportionately with AA doses between 0.2 and 1.5 g/kg, the values of these parameters did not increase much further at doses higher than 1.5 g/kg. Fixed-rate infusions at 0.6 g/min produced similar Cmax and AUC values with a 3-h infusion, and fixed-rate infusions at 0.8 g/min and 1 g/min resulted in slightly higher Cmax and AUC values than with the 3-h infusion. However, two patients complained about palpitation during the AA infusion due to the high-speed infusion rate. Therefore, a 3-h infusion is recommended for future studies.

A previous study has found that mutant KRAS or BRAF CRC cells exhibit high expression of GLUT1, leading to increased uptake of the oxidized form of vitamin C, dehydroascorbate (DHA). This increased DHA uptake causes oxidative stress as intracellular DHA is reduced to vitamin C, depleting glutathione and leading to energetic crisis and cell death. However, this phenomenon was only observed in KRAS and BRAF mutant cancer cells. No difference in efficacy was found between mCRC patients with wild-type RAS and mutant RAS or BRAF in our study. Therefore, not only patients with mutant RAS or BRAF but also those with wild-type RAS and BRAF should be tested in future studies. Of note, two patients with mutant BRAF responded well to treatment.

In preclinical studies, synergy has been demonstrated between AA and chemotherapy agents such as oxaliplatin [17]. Our group found that the combination of AA with chemotherapeutic agents, including oxaliplatin and irinotecan, achieved synergistic inhibitory effects in gastric cancer PDX models via an increase in oxidative stress [11]. Considering such synergistic effects, we modified the administration schedule of AA from a previous study [17], and AA was given once daily for three consecutive days in a 14-day cycle alongside mFOLFOX6 or FOLFIRI. Preclinical evidence suggests that apoptotic cell death occurs in many cancer cell lines exposed to AA concentrations > 5 mmol/L for < 1 h [16]. Comparable to that with AA monotherapy at 1.5 g/kg in the Caucasian population [17], our PK analysis revealed that AA at 1.5 g/kg sustained a mean plasma AA concentration exceeding 5 mmol/L for at least 5 h. Even though only half of the patients with mCRC received concomitant bevacizumab in this study, an objective response rate of 59.1% was achieved with the addition of AA to FOLFOX±bevacizumab as first-line therapy for mCRC, which compared favorably with that reported in previous trials of first-line FOLFOX plus bevacizumab. [25, 26] Considering its promising effect in mCRC and mGC, the additional efficacy of treatment when AA is combined with chemotherapy is worth investigating in larger clinical trials.

## Conclusions

In summary, the combination of AA at 1.5 g/kg once daily for three consecutive days with mFOLFOX6 or FOLFIRI with or without bevacizumab every 14 days exhibits a favorable safety profile and potential clinical efficacy in patients with mCRC or mGC. As a result, a randomized phase III study is ongoing investigating the additional efficacy of treatment when AA is combined with mFOLFOX6 ± bevacizumab as first-line therapy for patients with mCRC (NCT02969681).

## Additional files

Additional file 1: **Figure S1.** Evaluation of the ascorbic acid dose proportionality in part 1 of the study. The mean AA Cmax and AUC are presented vs increasing doses of AA (measured after the first administered dose of AA) on day 1 and day 3. AUC 0–9: area under the plasma concentration-time curve from time zero to hour 9; Cmax: maximum plasma concentration. (PDF 21 kb)

Additional file 2: **Figure S2.** Mean plasma concentration–time curve of ascorbic acid after fixed-rate infusions at 0.6, 0.8, 1.0 g/min to cancer patients. After i.v. administration, the plasma concentrations of ascorbic acid rose gradually and peaked at 3 h. Cmax, and AUC values of ascorbic acid display dose-dependent increases. Ascorbic acid concentrations in the high-dose groups remained at 10–20 mmol/L for more than 4 h and showed no accumulation in the body during the administrations. (PDF 20 kb)

Additional file 3: **Table S1.** Pharmacokinetic values. The volumes of distribution and clearance were similar to those parameters in the fixed infusion time group. When the recommended subsequent dose was administered, the plasma concentrations of AA were maintained at 10–20 mmol/L for more than 4 h. (DOCX 17 kb)

## Abbreviations

AA: Ascorbic acid
AUC: Area under the curve
CTCAE: Common terminology criteria for adverse events
DHA: Dehydroascorbate
DLT: Dose-limiting toxicity
ECOG: Eastern cooperative oncology group
mCRC: Metastatic colorectal cancer
mGC: Metastatic GC
MTD: Maximum tolerated dose
PDX: Patient-derived xenograft
PFS: Progression-free survival
PK: Pharmacokinetic
RECIST: Response Evaluation Criteria in Solid Tumors
RP2D: Recommended phase 2 dose
ULN: Upper limit of normal
