# Supplementary figures and images for: Phase I study of high-dose ascorbic acid with mFOLFOX6 or FOLFIRI in patients with metastatic colorectal cancer or gastric cancer
Source: BMC Cancer. 2019 May 16;19:460. doi: 10.1186/s12885-019-5696-z (PMC6524297; doi:10.1186/s12885-019-5696-z)

# Supplementary Figure 1

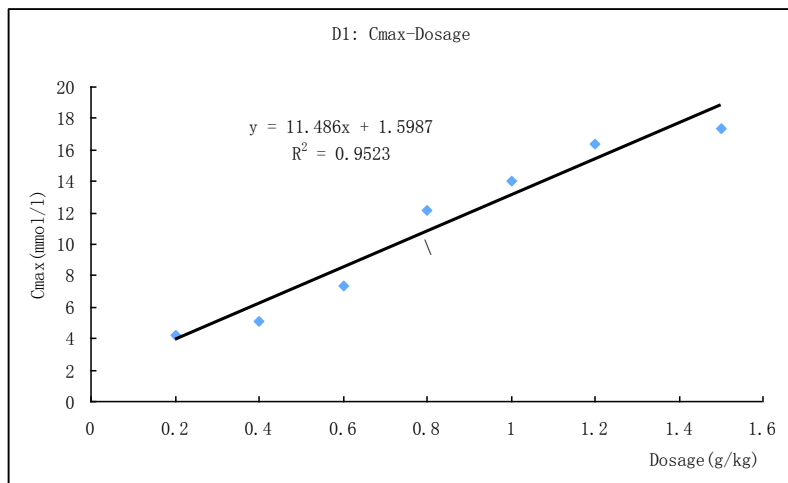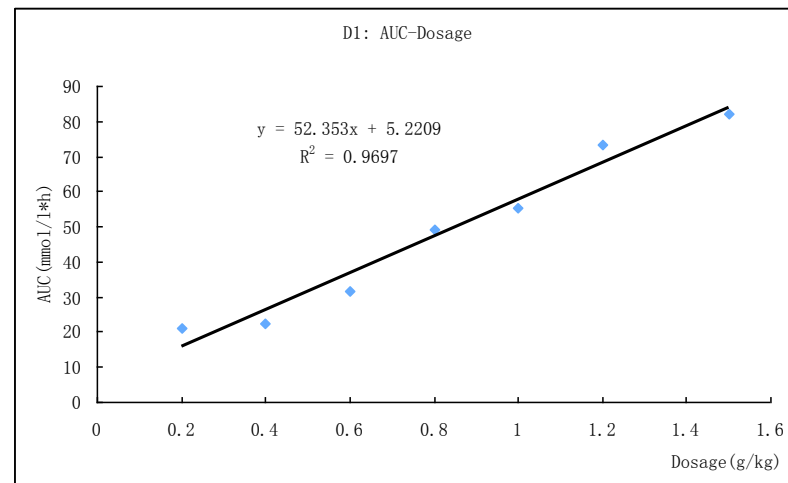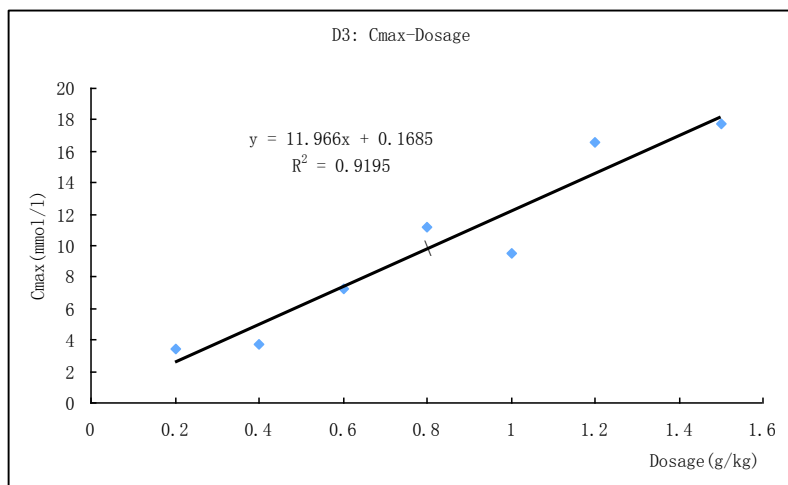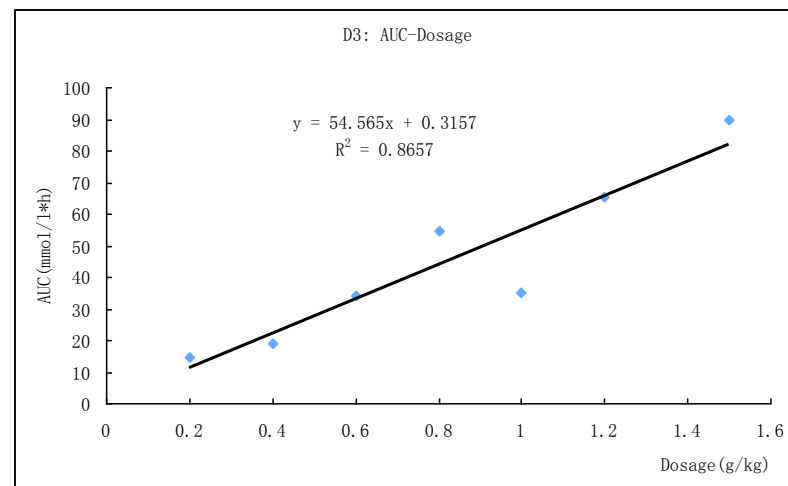

Supplement: Supplementary file 1 — Figure S1. Evaluation of the ascorbic acid dose proportionality in part 1 of the study. The mean AA Cmax and AUC are presented vs increasing doses of AA (measured after the first administered dose of AA) on day 1 and day 3. AUC 0–9: area under the plasma concentration-time curve from time zero to hour 9; Cmax: maximum plasma concentration. (PDF 21 kb) [file 12885_2019_5696_MOESM1_ESM.pdf]

Supplementary Figure 2

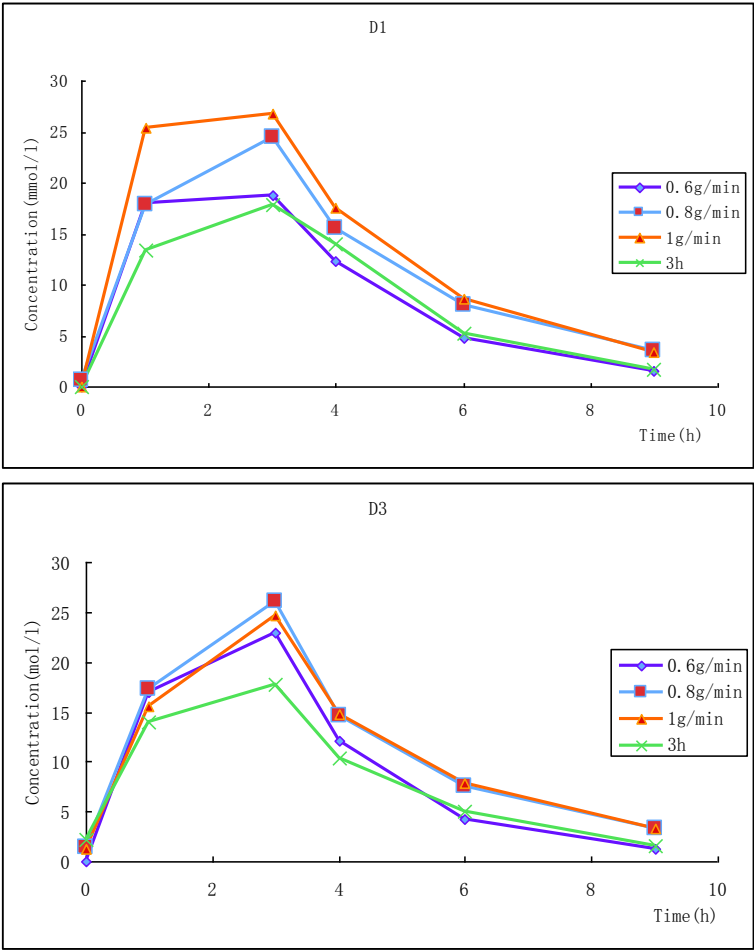

Supplement: Supplementary file 2 — Figure S2. Mean plasma concentration–time curve of ascorbic acid after fixed-rate infusions at 0.6, 0.8, 1.0 g/min to cancer patients. After i.v. administration, the plasma concentrations of ascorbic acid rose gradually and peaked at 3 h. Cmax, and AUC values of ascorbic acid display dose-dependent increases. Ascorbic acid concentrations in the high-dose groups remained at 10–20 mmol/L for more than 4 h and showed no accumulation in the body during the administrations. (PDF 20 kb) [file 12885_2019_5696_MOESM2_ESM.pdf]
