# Supplementary material for: Phase I study of high-dose ascorbic acid with mFOLFOX6 or FOLFIRI in patients with metastatic colorectal cancer or gastric cancer
Source: BMC Cancer. 2019 May 16;19:460. doi: 10.1186/s12885-019-5696-z (PMC6524297; doi:10.1186/s12885-019-5696-z)
Supplement: Supplementary file 3 — Table S1. Pharmacokinetic values. The volumes of distribution and clearance were similar to those parameters in the fixed infusion time group. When the recommended subsequent dose was administered, the plasma concentrations of AA were maintained at 10–20 mmol/L for more than 4 h. (DOCX 17 kb) [file 12885_2019_5696_MOESM3_ESM.docx]

**Supplementary Table 1 Pharmacokinetic values**

|  |  | *Unit* | *3h* | *0.6g/min* | *0.8g/min* | *1g/min* |
| --- | --- | --- | --- | --- | --- | --- |
| D1 | Cmax | mmol/l | 19.6±5.4 | 26.2±3.2 | 24.6±6.9 | 28.8±6.7 |
|  | t1/2_Lambda_z | h | 1.6±0.7 | 1.6±0.3 | 2.3±0.9 | 2.0±0.4 |
|  | AUCINF(observed) | mmol/l*h | 89.6±28.9 | 92.1±37.6 | 126.3±39.7 | 141.5±47.3 |
|  | Vz(observed) | ml | 13.1±5.3 | 12.6±4.0 | 14.1±3.1 | 11.2±3.2 |
|  | Cl(observed) | ml/h | 5.7±1.8 | 5.8±3.1 | 4.5±1.3 | 3.9±0.9 |
| D3 | Cmax | mmol/l | 18.4±6.0 | 22.9±6.9 | 26.4±5.8 | 24.6±9.2 |
|  | t1/2_Lambda_z | h | 1.7±0.6 | 1.5±0.3 | 2.3±0.8 | 2.3±0.5 |
|  | AUCINF(observed) | mmol/l*h | 85.1±41.3 | 93.4±12.8 | 123.8±33.6 | 119.0±41.0 |
|  | Vz(observed) | ml | 15.4±5.5 | 11.2±3.1 | 14.4±3.4 | 15.5±5.1 |
|  | Cl(observed) | ml/h | 6.5±2.4 | 5.0±0.7 | 4.5±1.1 | 4.6±1.0 |
